# Oral carbohydrate sensing enhances prefrontal cortex oxygenation, reduces perceived exertion, and improves high-intensity cycling performance: A randomized crossover trial

**DOI:** 10.1371/journal.pone.0349067

**Published:** 2026-05-12

**Authors:** Seung-Bo Park, Kyungjin Oh, Geonwoo Yang, Taenam Kim, Jea Woog Lee, Hyung-Jin Jeon, Doug Hyun Han

**Affiliations:** 1 Graduate School of Sports Medicine, CHA University, Seongnam, Republic of Korea; 2 Center for Translational Medicine, Medical Center Research Institute, CHA Bundang Medical Center, CHA University, Seongnam, Republic of Korea; 3 Department of Psychiatry, Chung-Ang University Hospital, Seoul, Republic of Korea; 4 College of Sports Science, Chung-Ang University, Anseong, Republic of Korea; University of Sao Paulo: Universidade de Sao Paulo, BRAZIL

## Abstract

**Purpose:**

Carbohydrate mouth rinsing (CHO-MR) and music listening (MUS) are non-metabolic interventions proposed to attenuate cognitive and perceptual fatigue during exercise. However, their comparative effects on prefrontal cortical oxygenation, executive function, and perceived exertion during high-intensity endurance performance remain unclear. This study examined the effects of CHO-MR and MUS on dorsolateral prefrontal cortex (DLPFC) oxygenation, cognitive performance, perceived exertion, and cycling performance during a 4-km time trial (TT).

**Methods:**

Eleven trained cyclists (7 men, 4 women) completed a randomized, single-blind, crossover trial under three conditions: CHO-MR, MUS (120 beats/min), and placebo mouth rinse (PLA). Bilateral DLPFC oxygenation was assessed using functional near-infrared spectroscopy at rest, during Stroop testing, post-intervention, and post-TT. Stroop performance, rating of perceived exertion (RPE); every 500 m, completion time, power output, heart rate, and blood lactate were measured. Outcomes were analyzed using generalized estimating equations adjusted for period, sequence, and first-order carryover effects with robust standard errors.

**Results:**

CHO-MR increased bilateral DLPFC oxygenation compared with MUS and PLA at post-intervention and post-TT time points (*p* < 0.05), with no differences at rest or baseline. Stroop performance was superior in CHO-MR relative to MUS and PLA following the TT (*p* < 0.001), without between-condition differences at earlier assessments. RPE was lower in CHO-MR than PLA across all intervals (*p* ≤ 0.01) and lower than MUS at mid-trial distances (*p* < 0.05). CHO-MR resulted in faster completion times, greater mean power output, and higher mean speed compared with MUS and/or PLA (*p* < 0.05), whereas peak power, heart rate, and blood lactate did not differ among conditions. Pooled change-score analyses demonstrated positive associations between bilateral ΔDLPFC oxygenation (*p* < 0.001) and between ΔDLPFC oxygenation and ΔStroop performance (*p* < 0.05).

**Conclusion:**

CHO-MR enhances bilateral DLPFC oxygenation, preserves executive function, and reduces perceived exertion during high-intensity endurance exercise, translating into improved performance without detectable peripheral metabolic alterations. These findings support a central neurocognitive mechanism underlying the ergogenic effects of oral carbohydrate sensing.

**Trial registration:**

ClinicalTrials.gov NCT07099807

## Introduction

Carbohydrate mouth rinsing (CHO-MR) enhances exercise performance primarily through central mechanisms, rather than peripheral substrate provision [1,2]. Activation of oral carbohydrate receptors stimulates reward- and control-related brain regions, including the dorsolateral prefrontal cortex (DLPFC) and striatum, without altering circulating fuel availability [3,4]. This central activation has been associated with reductions in perceived exertion and preservation of cognitive function during exercise [5,6]. Music listening (MUS) represents another non-metabolic intervention capable of modulating exercise performance via central pathways. By engaging prefrontal cortex and mesolimbic dopaminergic networks, music can elevate affect, influence arousal, and attenuate ratings of perceived exertion (RPE) during sustained or high-intensity exercise [7,8]. Although CHO-MR and MUS differ mechanistically, both may attenuate fatigue-related decrements in DLPFC function by influencing central processes governing motivation, attentional control, and effort perception [9,10].

Despite this shared central orientation, the interventions are neurophysiologically distinct. CHO-MR primarily activates gustatory and reward-related pathways and is considered a relatively focal neuromodulatory stimulus, with minimal direct cardiometabolic effects. In contrast, MUS is multidimensional, eliciting concurrent psychological (e.g., affect, arousal), physiological (e.g., autonomic activity), and psychophysical responses [11,12]. These differences suggest that CHO-MR may exert more targeted modulation of cortical regions subserving cognitive control, whereas MUS may induce broader psychophysiological regulation during exercise [3,13–15]. Furthermore, the ergogenic effects of music may diminish in highly trained or fatigued individuals, potentially limiting its effectiveness under near-maximal conditions [9,16,17]. CHO-MR may therefore be particularly suitable when auditory stimulation is restricted (e.g., competition settings) or when targeted prefrontal modulation is desired without altering arousal. Conversely, MUS may be more advantageous during longer-duration or submaximal exercise where affective modulation plays a greater role, although its benefits appear less consistent under high-intensity or fatigued conditions [9,17]. High-intensity endurance exercise imposes substantial central demands related to effort regulation, motivation, and attentional control, processes in which the DLPFC plays a key integrative role. Additionally, high-intensity exercise is associated with variable hemodynamic responses in the DLPFC, and altered prefrontal cortical activity under such conditions has been linked to elevated perceived exertion and impaired executive performance [18,19]. Accordingly, interventions that sustain DLPFC activation during strenuous exercise may mitigate cognitive–perceptual strain and improve performance regulation.

To isolate these central effects under controlled conditions, we employed a 4-km cycling time trial (TT), a near-maximal effort lasting approximately 5–6 min. This model imposes pronounced cognitive and motivational demands while minimizing the influence of exogenous fuel availability, thereby facilitating examination of neurophysiological modulation independent of metabolic supplementation [9,10,19]. Although such short-duration efforts may not necessarily induce classical central fatigue defined by reduced voluntary activation, they provide a sensitive paradigm for assessing cortical oxygenation and perceptual responses under high effort conditions.

Despite extensive independent investigation of CHO-MR and MUS, direct comparisons of their effects on cerebral oxygenation and DLPFC-dependent cognitive performance during high-intensity endurance exercise remain scarce. Therefore, the purpose of this study was to examine the effects of CHO-MR and MUS on bilateral DLPFC oxygenation, executive function, perceived exertion, and cycling performance during a 4-km time trial using a randomized crossover design. We tested the hypothesis that: (1) both CHO-MR and MUS would increase DLPFC oxygenation during a cognitive task relative to placebo (PLA); (2) both interventions would improve executive performance compared with PLA; and (3) CHO-MR would sustain greater DLPFC oxygenation during subsequent high-intensity exercise than MUS, indicating differential central modulation independent of peripheral metabolic alterations.

## Methods

### Participants

Eleven well-trained cyclists (7 men and 4 women) participated in this randomized crossover trial. All participants engaged in structured cycling training ≥5 days/week, with a mean training volume of approximately 4 h/day. Participant characteristics are summarized in Table 1. Eligibility criteria included: (1) ≥2 years of endurance cycling training, (2) absence of cardiovascular, neurological, or metabolic disorders, and (3) no use of medications known to affect cardiovascular or cognitive function. Participants provided written informed consent prior to enrollment.

**Table 1:** Participant main characteristics and performance details (n = 11).

Variable	Mean ± SD
Age (years)	20.82 ± 0.92
Body mass (kg)	71.00 ± 10.48
Height (cm)	170.71 ± 9.25
Functional threshold power (W)	252.00 ± 64.29
VO2max (mL/kg/min)	58.35 ± 8.85
Lactate threshold (W)	102.43 ± 36.60
vLamax (mmol/L/s)	0.99 ± 0.16
Peak lactate (mmol/L)	14.19 ± 2.46

Values are presented as mean ± SD. SD, standard deviation; VO2max, maximal oxygen uptake consumption; vLamax, maximal lactate production rate; peak La, peak lactate.

The study protocol was approved by the Institutional Review Board of Chung-Ang University (approval number: 1041078–20250131-BR-024) and conducted in accordance with the Declaration of Helsinki. Participants were recruited between April 17 and April 27, 2025. The trial was registered retrospectively. All ongoing and related trials for this intervention have been registered. The original protocol targeted 15 participants; however, only 11 completed the study due to participant withdrawal and scheduling constraints. A mental fatigue task originally planned prior to intervention was omitted to reduce participant burden. The primary outcome was regional cerebral oxygenation measured via functional near-infrared spectroscopy (fNIRS). Planned analyses of functional connectivity were not performed due to technical limitations during data acquisition. These protocol modifications were finalized prior to statistical analysis.

### Preliminary assessments

Anthropometric measurements, including body mass and stature, were obtained using standardized procedures prior to exercise testing. Participants subsequently completed an incremental cycling test on a calibrated Wattbike Pro ergometer (Wattbike Ltd., Nottingham, UK) to determine functional threshold power (FTP). The test was preceded by a standardized warm-up consisting of 5 min of cycling at a self-selected moderate intensity, followed by 3 min at approximately 50% of the anticipated maximal workload. The incremental protocol began at 100 W, with workload increased by 15 W/min for female cyclists and 20 W/min for male cyclists, consistent with previously validated ramp-testing methodologies in trained cyclists [20,21]. Participants were instructed to maintain a cadence ≥60 revolutions per minute (rpm) and were encouraged to exercise to volitional exhaustion. The test was terminated when cadence fell below 60 rpm despite verbal encouragement. FTP was estimated as 75% of the peak power output (PPO) achieved during the final completed stage of the ramp test [22,23]. Accordingly, no fixed power target was prescribed for the 4-km TT; participants were informed of their individually determined FTP as a reference for initial pacing and were instructed to complete each trial as an all-out effort using self-selected pacing, which corresponded to approximately 105–110% of FTP.

### Study design

**Fig 1:**
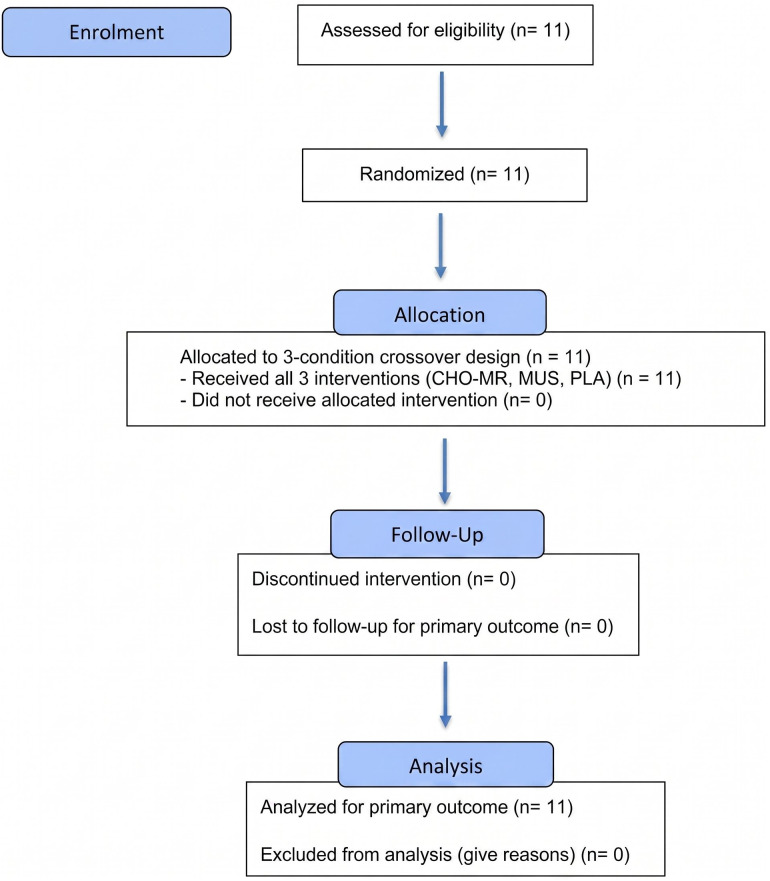
Flowchart for the randomized crossover trial. Eleven participants were assessed for eligibility, enrolled, and randomized to complete three experimental conditions (CHO-MR, MUS, and PLA) in a counterbalanced crossover design. All participants completed all conditions, and all were included in the final analysis. CHO-MR, carbohydrate mouth rinse; MUS, music listening; PLA, placebo mouth rinse.

This study employed a single-blind, randomized, counterbalanced crossover design comprising five laboratory visits (Fig 1). During the initial visit (Visit 1), participants completed medical screening, anthropometric assessments, and an incremental cycling test to determine FTP. The second visit (Visit 2) consisted of a full familiarization session, including a 4-km cycling time trial (TT), mouth-rinsing procedures, cognitive testing, and physiological monitoring to minimize learning effects. Visits 3–5 comprised the three experimental trials performed under the following conditions: carbohydrate mouth rinse (CHO-MR; with maltodextrin, music listening (MUS), and placebo mouth rinse (PLA; with an artificial sweetener). The order of conditions was randomized and counterbalanced using a computer-generated allocation sequence to minimize sequence effects. A washout period of 3–7 days separated experimental trials to reduce residual fatigue and potential carryover effects. All sessions were conducted at the same time of the day for each participant to control for circadian influences. The primary outcome was bilateral DLPFC oxygenation measured via fNIRS.

### Experimental protocol

**Fig 2:**
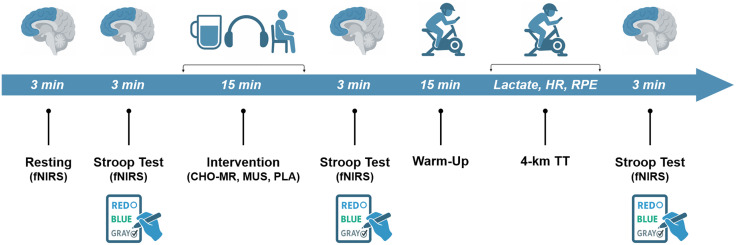
Schematic overview of the experimental protocol. Resting-state fNIRS recordings and Stroop tasks (3 min each) were performed to assess DLPFC oxygenation before intervention, immediately after intervention (CHO-MR, MUS, or PLA), and following completion of the 4-km cycling time trial. Blood lactate concentration, heart rate, and perceived exertion were recorded at predefined intervals. *Abbreviations*: CHO-MR, carbohydrate mouth rinsing; DLPFC, dorsolateral prefrontal cortex; fNIRS, functional near-infrared spectroscopy; HR, heart rate; MUS, music listening; PLA, placebo mouth rinse; [La⁻], blood lactate concentration; RPE, rating of perceived exertion; TT, time trial.

Each experimental session followed an identical sequence (Fig 2). Upon arrival, participants rested in a seated position for 3-min during which resting-state DLPFC oxygenation was recorded using fNIRS. This was immediately followed by a 3-min fNIRS acquisition during performance of a computerized Stroop test to assess baseline task-related cortical activation. Participants then underwent a 15-min intervention consisting of CHO-MR, MUS, or PLA according to the randomized crossover allocation. Immediately following the intervention, a second 3-min Stroop task was performed with concurrent fNIRS measurement to assess intervention-induced changes in DLPFC oxygen. Capillary blood lactate concentrations ([La⁻]) was measured in the seated position prior to warm-up. Participants subsequently performed a standardized warm-up consisting of 10 min of cycling at 60% of maximal oxygen uptake (VO₂max), followed by 5 min of passive recovery. Pre-exercise [La⁻] was reassessed immediately before the 4-km TT. During the TT, heart rate (HR) was recorded continuously and RPE was obtained at 500-m intervals. Capillary [La⁻] was measured immediately upon completion of the TT. Finally, a fourth 3-min fNIRS acquisition was conducted during a Stroop task to evaluate post-exercise DLPFC oxygenation.

### Carbohydrate mouth rinse

The CHO-MR solution was prepared by dissolving maltodextrin powder (Nutricost LLC, Vineyard, UT, USA) in distilled water to yield a 6.4% solution (64 g/L). During each experimental session, participants completed five standardized rinses of 25 mL each. For each rinse, the solution was swilled throughout the oral cavity for 10 s before being expectorated into a collection container. Rinses were separated by 30-s intervals. The PLA solution consisted of distilled water containing a minimal concentration (approximately 0.05 g/L) of non-caloric sucralose (Shaanxi Hongda Phytochemistry Co., Ltd., Xi’an, China) to mimic the subtle sweetness of the maltodextrin solution. To preserve blinding, both solutions were colorless and presented in opaque containers. Participants were informed that all rinses could contain carbohydrate but were not informed of the specific condition administered in each trial. Participants were instructed not to swallow any solution. Rinse volume, duration, and frequency were identical between CHO-MR and PLA conditions to standardize oral sensory exposure.

### Music intervention

MUS consisted of high-tempo music (120 beats per minute; bpm) selected from a standardized publicly available playlist (“120 BPM Best Dance Music for Running and Working Out,” YouTube). Music was delivered for 15 min via standardized in-ear earbuds connected to a smartphone device. Sound intensity was calibrated to approximately 65 dB (≈50% of maximum device output) using manufacturer specifications to ensure consistent auditory exposure. The selected tempo (120 bpm) was chosen based on prior literature demonstrating modulation of psychophysiological responses and perceived exertion during exercise [24,25]. Music selection, and playback device, and sound intensity were standardized across participants. Owing to the perceptible nature of the music condition, full double-blinding across all experimental conditions was not feasible.

### Stroop test

Executive function was assessed using a computerized Stroop color–word task administered on a Windows-based tablet. The 3-min Stroop task was used as a brief probe of executive function with concurrent fNIRS measurement, not as a mental-fatigue induction protocol; therefore, subjective mental fatigue ratings were not collected. Assessments were performed at three time points: pre-intervention (baseline), immediately post-intervention, and post-TT. Each 3-min Stroop task included both congruent (word meaning matched ink color) and incongruent (word meaning differed from ink color) stimuli. Participants were instructed to identify the ink color while inhibiting the automatic tendency to read the word. Testing was conducted in a seated position within a controlled environment to minimize external distractions. All participants completed a familiarization session prior to experimental trials to reduce learning effects. Stroop performance metrics (e.g., reaction time and/or accuracy) were recorded for subsequent analysis.

### 4-km cycling time trial

Participants performed a TT on a calibrated Wattbike Pro ergometer. They were instructed to complete the distance as quickly as possible. Initial pacing guidance suggested maintaining approximately 105–110% of previously determined FTP, although pacing was ultimately self-selected. Real-time feedback on distance completed, instantaneous power output (W), and elapsed time was displayed throughout the trial. Standardized verbal encouragement was provided. Performance outcomes included completion time (primary performance variable), mean power output, and mean speed. RPE was recorded at 500-m intervals using the Borg 6–20 scale [26]. Capillary [La^-^] was measured 5 min after warm-up (pre-exercise) and immediately following the TT (post-exercise). For each assessment, 20 µL of capillary blood was obtained from the earlobe and analyzed using an amperometric–enzymatic method (Biosen C-line, EKF Diagnostics GmbH, Barleben, Germany). HR was monitored continuously using a Polar H10 sensor (Polar Electro, Kempele, Finland), with values recorded at 500-m intervals.

### Assessment of hemodynamic changes in the prefrontal cortex

**Fig 3:**
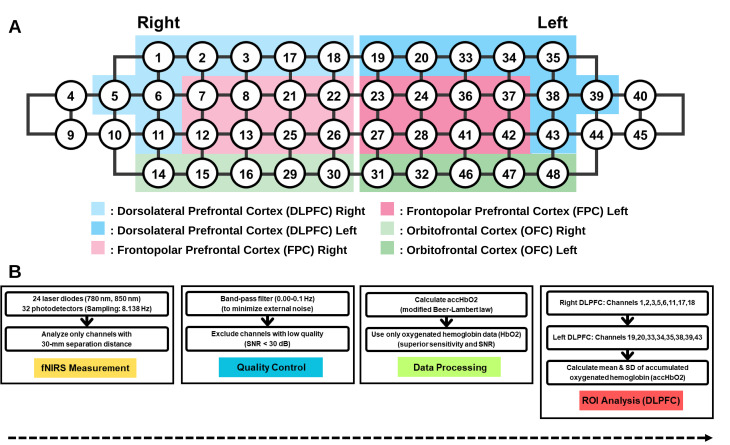
Region-of-interest (ROI) configuration and fNIRS processing workflow. (A) Channel layout illustrating bilateral DLPFC regions analyzed in the primary outcome. The right DLPFC comprised channels 1, 2, 3, 5, 6, 11, 17, and 18, while the left DLPFC included channels 19, 20, 33, 34, 35, 38, 39, and 43. (B) Preprocessing workflow. Only channels with 30-mm source–detector separation were included. Signals were band-pass filtered (0.01–0.10 Hz), low-quality channels (signal-to-noise ratio < 30 dB) were excluded and accHbO₂ concentration changes were computed using the modified Beer–Lambert law. accHbO₂, accumulated oxygenated hemoglobin; DLPFC, dorsolateral prefrontal cortex; FPC, frontopolar cortex; fNIRS, functional near-infrared spectroscopy; HbO, oxygenated hemoglobin; HbR, deoxygenated hemoglobin; OFC, orbitofrontal cortex; ROI, region of interest; VLPFC, ventrolateral prefrontal cortex.

Cortical hemodynamic response were assessed using a high-density fNIRS system (NIRSIT; OBELAB Inc., Seoul, Korea). The device consisted of 24 light sources emitting at dual wavelengths (780 and 850 nm) and 32 photodetectors, sampled at 8.138 Hz. The source–detector separations ranged from 15 to 30 mm. Only channels with a 30-mm separation were included in the primary analysis to maximize cortical sensitivity. Raw optical density signals were converted to concentration changes in oxygenated hemoglobin (HbO) and deoxygenated hemoglobin (HbR) using the modified Beer–Lambert law. Signals were band-pass filtered (0.01–0.10 Hz) to attenuate slow signal drift and high-frequency noise associated with motion and physiological artifacts. Channels with poor signal quality (signal-to-noise ratio < 30 dB) were excluded prior to analysis. Prefrontal cortex activation was assessed based on the mean change in oxygenated hemoglobin concentration (ΔHbO) at each time point relative to the resting baseline. Although both HbO and HbR signals were available, analyses focused on HbO due to its greater signal-to-noise ratio and sensitivity to task-related cortical activation in prior fNIRS literature [27]. Mean HbO concentration changes were calculated for predefined regions of interest (ROIs). Eight frontal ROIs were initially identified: right and left DLPFC, right and left frontopolar cortex (FPC), right and left ventrolateral prefrontal cortex (VLPFC,) and right and left orbitofrontal cortex (OFC). Channel assignments for each ROI are presented in Fig 3. Consistent with the study hypothesis, primary analyses focused on bilateral DLPFC.

### Statistical analyses

All statistical analyses were performed using Python (v3.13.3) with the *statsmodels* (v0.14.5) and *SciPy* (v1.15.3) packages. Data are presented as mean ± standard deviation (SD) unless otherwise specified; 95% confidence intervals (CI) are reported where appropriate. Statistical significance was set at α = 0.05 (two-tailed). The primary outcome was bilateral DLPFC oxygenated hemoglobin concentration change (ΔaccHbO₂). Repeated measures collected across experimental conditions and stages were analyzed using generalized estimating equations (GEE) with a Gaussian family, exchangeable working correlation structure, and robust (sandwich) standard errors to account for within-subject dependence in the crossover design. For primary analyses, models included fixed effects for condition (CHO-MR, MUS, PLA), stage (resting, baseline, post-intervention, post–time trial), and their interaction (condition × stage). To account for crossover-related effects, models were adjusted for period (modeled as a numeric term), sequence, and first-order carryover (previous condition). Global robust Wald χ² tests were used to evaluate main effects and interactions. When significant effects were detected, estimated marginal means (EMMs) were derived and pairwise comparisons were performed with Holm correction for multiple testing. Cycling performance outcomes (completion time, mean power, peak power, mean speed) were analyzed using analogous GEE models with condition as the primary predictor and adjustment for period, sequence, and first-order carryover. RPE, collected at 500-m intervals during the time trial, was analyzed using GEE models with distance treated as the repeated stage factor. Pairwise contrasts at each distance were evaluated using EMMs with Holm adjustment. Associations between post-exercise changes in DLPFC ΔaccHbO₂ and changes in Stroop performance (ΔStroop) were examined using GEE regression models adjusted for period, sequence, and first-order carryover, with robust standard errors.

### Sample size and power analysis

An a priori sample-size estimation was conducted using G*Power 3.1 (Heinrich Heine University Düsseldorf, Düsseldorf, Germany) based on a repeated-measures analysis of variance (ANOVA) framework (three within-subject conditions), which served as a design-stage approximation prior to adoption of the final GEE-based model. Assuming effect size f = 0.36, α = 0.05, power (1 − β) = 0.80, correlation among repeated measures r = 0.50, and ε = 1.0, the required sample size was N = 14 completers; therefore, N = 15 was targeted to account for potential attrition.

Given the final analytic approach using GEE with crossover adjustment, statistical power was additionally evaluated via Monte Carlo simulation (3,000 replicates) matched to the observed data structure (11 participants, three conditions, four stages). Power was estimated for: (i) the global condition × stage interaction and (ii) the Holm-adjusted CHO-MR versus PLA contrast at post-TT. Estimated power ranged from 0.67–0.92 across hemispheres and testing criteria.

## Results

### Changes in DLPFC across conditions

**Fig 4:**
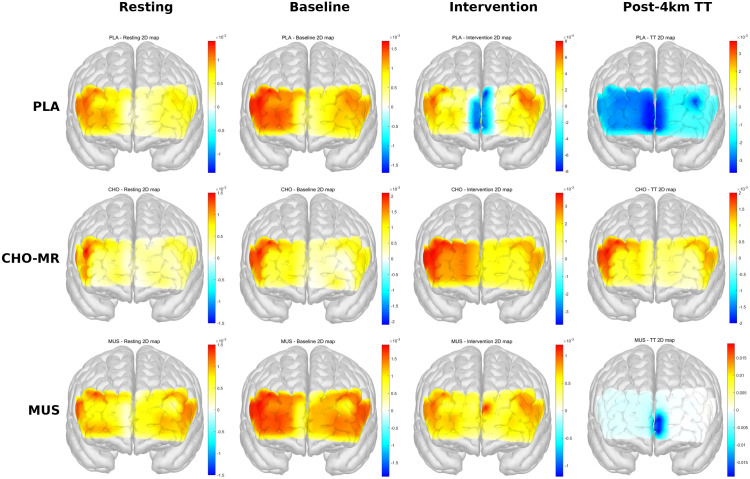
Topographic 2D maps of prefrontal cortex oxygenated hemoglobin concentration changes (ΔHbO) across experimental conditions and time points.

**Fig 5:**
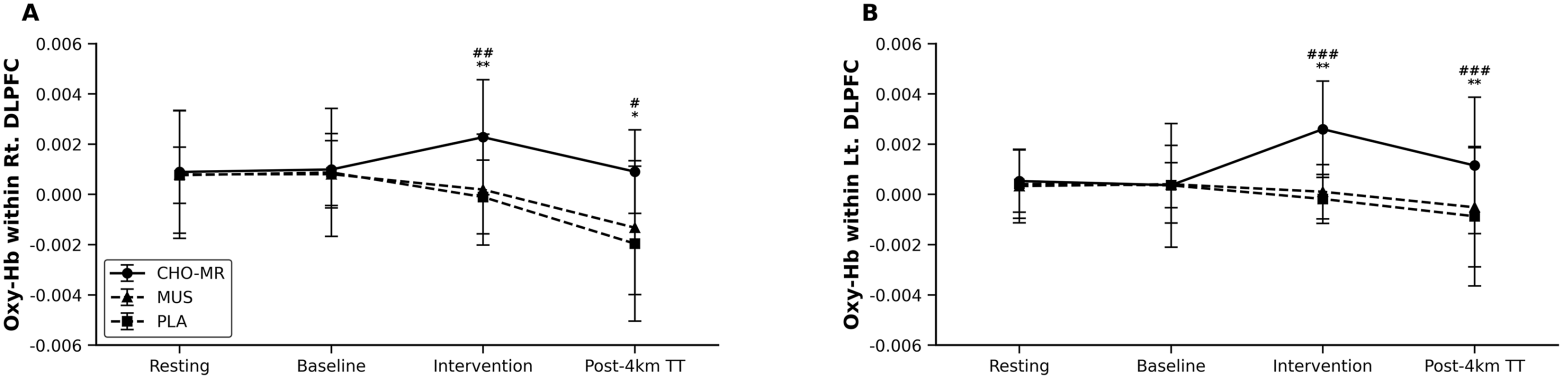
Changes in oxygenated hemoglobin (HbO) within the DLPFC across experimental conditions. (A) Right DLPFC and (B) left HbO concentration changes across rest, baseline (task), post-intervention, and post-TT under CHO-MR, MUS, and PLA conditions. Global model results are shown for stage and condition × stage interaction. Values are presented as mean ± SD. Between-condition comparisons within stages were conducted using Holm-adjusted EMM. Symbols denote significant differences between CHO-MR and PLA (* p < 0.05, ** p < 0.01, *** p < 0.001) and between CHO-MR and MUS (p < 0.05, ## p < 0.01, ### p < 0.001). HbO, oxygenated hemoglobin; CHO-MR, carbohydrate mouth rinsing; DLPFC, dorsolateral prefrontal cortex; MUS, music listening; PLA, placebo mouth rinse; TT, 4-km cycling time trial.

The spatial distribution of ΔHbO across the prefrontal cortex under each experimental condition and time point is presented in Fig 4. The topographic maps illustrate that CHO-MR was associated with sustained or increased prefrontal oxygenation at both the Intervention and Post-4 km TT stages, whereas PLA and MUS exhibited progressive reductions in ΔHbO following the time trial. These maps are presented for illustrative purposes to depict the spatial pattern of cortical hemodynamic responses; quantitative statistical analyses of bilateral DLPFC ΔHbO are reported below (Fig 5).

Maps represent the spatial distribution of mean ΔHbO (mM) across all fNIRS channels in the prefrontal cortex for each condition (PLA, CHO-MR, MUS) at four time points: Resting, Baseline, Post-intervention, and Post-4 km TT. Warm colors (red–yellow) indicate increases in ΔHbO relative to the resting baseline, whereas cool colors (blue) indicate decreases. Color scale bars represent the range of ΔHbO values (×10 ⁻ ³ mM) and are independently scaled for each panel. Maps are presented for illustrative purposes to depict the spatial distribution of prefrontal hemodynamic responses; quantitative analyses are reported in Fig 5.

CHO-MR, carbohydrate mouth rinse; DLPFC, dorsolateral prefrontal cortex; fNIRS, functional near-infrared spectroscopy; MUS, music listening; PLA, placebo; ΔHbO, change in oxygenated hemoglobin concentration.

### Right DLPFC

For the right DLPFC, there was a significant main effect of stage on ΔaccHbO₂ (robust Wald χ²(3) = 14.94, *p* < 0.01) and a significant condition × stage interaction (χ²(6) = 13.52, *p* < 0.05), whereas the main effect of condition was not significant (χ²(2) = 0.04, *p* ≥ 0.05; Fig 5A). Holm-adjusted EMM-based pairwise comparisons indicated no between-condition differences at resting or baseline (all *p* ≥ 0.05). Immediately post-intervention, ΔaccHbO₂ was greater in CHO-MR compared with PLA (z = 3.40, *p* < 0.01) and MUS (z = 3.21, *p* < 0.01), with no difference between PLA and MUS (*p* ≥ 0.05). Following the 4-km time trial (post-TT), ΔaccHbO₂ remained greater in CHO-MR than in PLA (z = 2.60, *p* < 0.05) and MUS (z = 2.46, *p* < 0.05), whereas PLA and MUS did not differ (*p* ≥ 0.05; Fig 5A).

### Left DLPFC

For the left DLPFC, the condition × stage interaction was significant (χ²(6) = 31.75, *p* < 0.001), whereas the main effects of condition (χ²(2) = 1.29, *p* ≥ 0.05) and stage (χ²(3) = 5.86, *p* ≥ 0.05) were not significant (Fig 5B). As observed in the right hemisphere, no between-condition differences were present at resting or baseline. Post-intervention, ΔaccHbO₂ was greater in CHO-MR compared with PLA (z = 3.30, *p* < 0.01) and MUS (z = 4.88, *p* < 0.001), with no difference between PLA and MUS (*p* ≥ 0.05). This pattern persisted post-TT with CHO-MR exceeding PLA (z = 3.35, *p* < 0.01) and MUS (z = 4.29, *p* < 0.001), while PLA and MUS remained comparable (*p* ≥ 0.05; Fig 5B).

### Changes in Stroop performance across conditions

**Fig 6:**
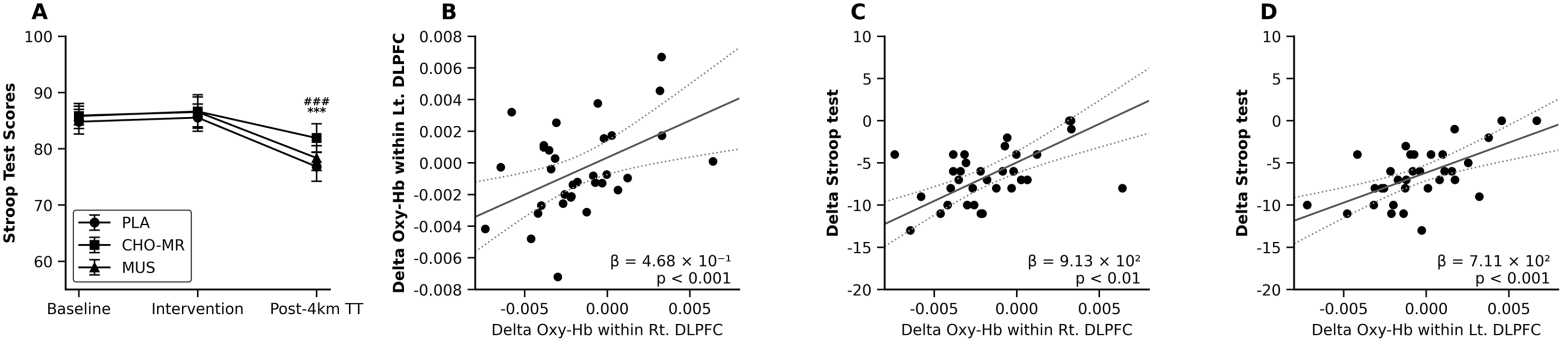
Stroop performance and associations with DLPFC oxygenation. (A) Stroop performance across baseline, intervention, and post-TT under PLA, CHO-MR, and MUS conditions. (B–D) GEE-based associations using pooled change scores (Δ = post-TT − baseline; n = 33): (B) right versus left DLPFC ΔaccHbO₂, (C) ΔStroop versus right DLPFC ΔaccHbO₂, and (D) ΔStroop versus left DLPFC ΔaccHbO₂. Values are mean ± SD. In panel (A), symbols indicate Holm-adjusted pairwise differences at post-TT (* *p* < 0.05, ** *p* < 0.01, *** *p* < 0.001 for CHO-MR vs. PLA; # *p* < 0.05, ## *p* < 0.01, ### *p* < 0.001 for CHO-MR vs. MUS). ΔaccHbO₂, accumulated oxygenated hemoglobin change; CHO-MR, carbohydrate mouth rinsing; DLPFC, dorsolateral prefrontal cortex; GEE, generalized estimating equations; HbO, oxygenated hemoglobin; MUS, music listening; PLA, placebo mouth rinse; TT, time trial; Δ, change from baseline to post-TT.

Stroop performance demonstrated a significant main effect of stage (robust Wald χ²(2) = 122.82, *p* < 0.001) and a significant condition × stage interaction (χ²(4) = 51.33, *p* < 0.001), whereas the main effect of condition was not significant (χ²(2) = 4.37, *p* ≥ 0.05; Fig 6A). Holm-adjusted pairwise comparisons revealed no between-condition differences at baseline or post-intervention (all *p* ≥ 0.05). However, post-TT, Stroop scores were higher in the CHO-MR condition compared with both PLA and MUS (both *p* < 0.001), whereas PLA and MUS did not differ (*p* ≥ 0.05; Fig 6A).

### Associations between ΔaccHbO2 changes and Stroop performances

Associations were examined using pooled change scores (Δ = post-TT − baseline) across all conditions (n = 33 observations), with GEE models adjusted for period, sequence, and first-order carryover. Changes in ΔaccHbO₂ within the right DLPFC were positively associated with ΔaccHbO₂ within the left DLPFC (β = 0.468, *p* < 0.001; Fig 6B). Additionally, improvements in Stroop performance (ΔStroop) were positively associated with ΔaccHbO₂ in the right DLPFC (β = 913, *p* < 0.01; Fig 6C) and in the left DLPFC (β = 711, *p* < 0.001; Fig 6D).

### Performance outcomes during the 4-km time trial

**Fig 7:**
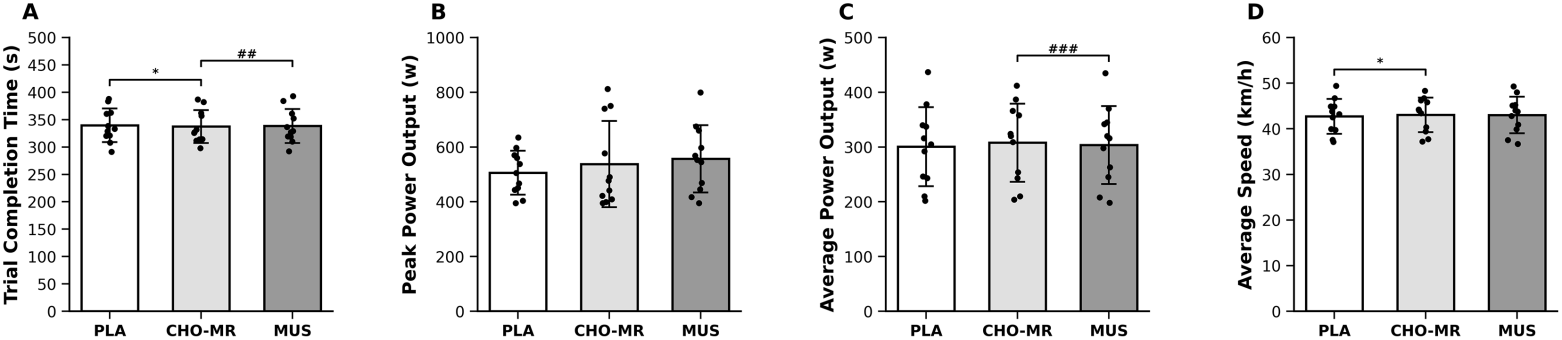
Effects of CHO-MR, MUS, and PLA on performance outcomes during the 4-km cycling time trial. (A) Completion time (s). (B) Peak power (W). (C) Mean power output (W). (D) Mean speed (km/h). Analyses were conducted using GEE adjusted for period, sequence, and first-order carryover. Symbols denote Holm-adjusted pairwise differences (* *p* < 0.05, ** *p* < 0.01, *** *p* < 0.001 for CHO-MR vs. PLA; # *p* < 0.05, ## *p* < 0.01, ### *p* < 0.001 for CHO-MR vs. MUS). Values are presented as mean ± SD with individual data points overlaid. *Abbreviations*: CHO-MR, carbohydrate mouth rinsing; GEE, generalized estimating equations; MUS, music listening; PLA, placebo mouth rinse.

Cycling performance differed between experimental conditions (CHO-MR, MUS, and PLA) when analyzed using GEE models adjusted for period, sequence, and first-order carryover, with robust standard errors (Fig 7A–D). A significant main effect of condition was observed for completion time, mean power output, and average speed (all *p* < 0.05), whereas peak power did not differ between conditions (*p* ≥ 0.05). Mean completion times were 336.64 ± 7.30 s (CHO-MR), 339.43 ± 7.40 s (MUS), and 339.36 ± 7.50 s (PLA), corresponding to a −0.80% difference between CHO-MR and PLA, and a + 0.02% difference between MUS and PLA. Holm-adjusted pairwise comparisons indicated that completion time was shorter in the CHO-MR condition compared with PLA (*p* < 0.05) and MUS (*p* < 0.01). Mean power output was greater in CHO-MR compared with MUS (*p* < 0.001), with no significant difference between CHO-MR and PLA (all *p* ≥ 0.05). Mean speed was higher in CHO-MR compared with PLA (*p* < 0.05), whereas other pairwise contrasts were not significant (*p* ≥ 0.05).

### Ratings of perceived exertion

RPE during the 4-km time trial demonstrated significant main effects of condition (*p* < 0.01) and distance (*p* < 0.001), as well as a significant condition × distance interaction (*p* < 0.001; Fig 7A). Holm-adjusted pairwise comparisons indicated that RPE was lower in the CHO-MR condition compared with PLA at all distance intervals (500 m: *p* < 0.01; 1000–4000 m: *p* < 0.001, except 2500 m: *p* < 0.05). Compared with MUS, CHO-MR elicited lower RPE from 1000 to 3000 m (1000 m: *p* < 0.05; 1500–3000 m: *p* < 0.001). No differences were observed between PLA and MUS at any distance interval (all *p* ≥ 0.05).

### Physiological parameters

#### Blood lactate concentration.

**Fig 8:**
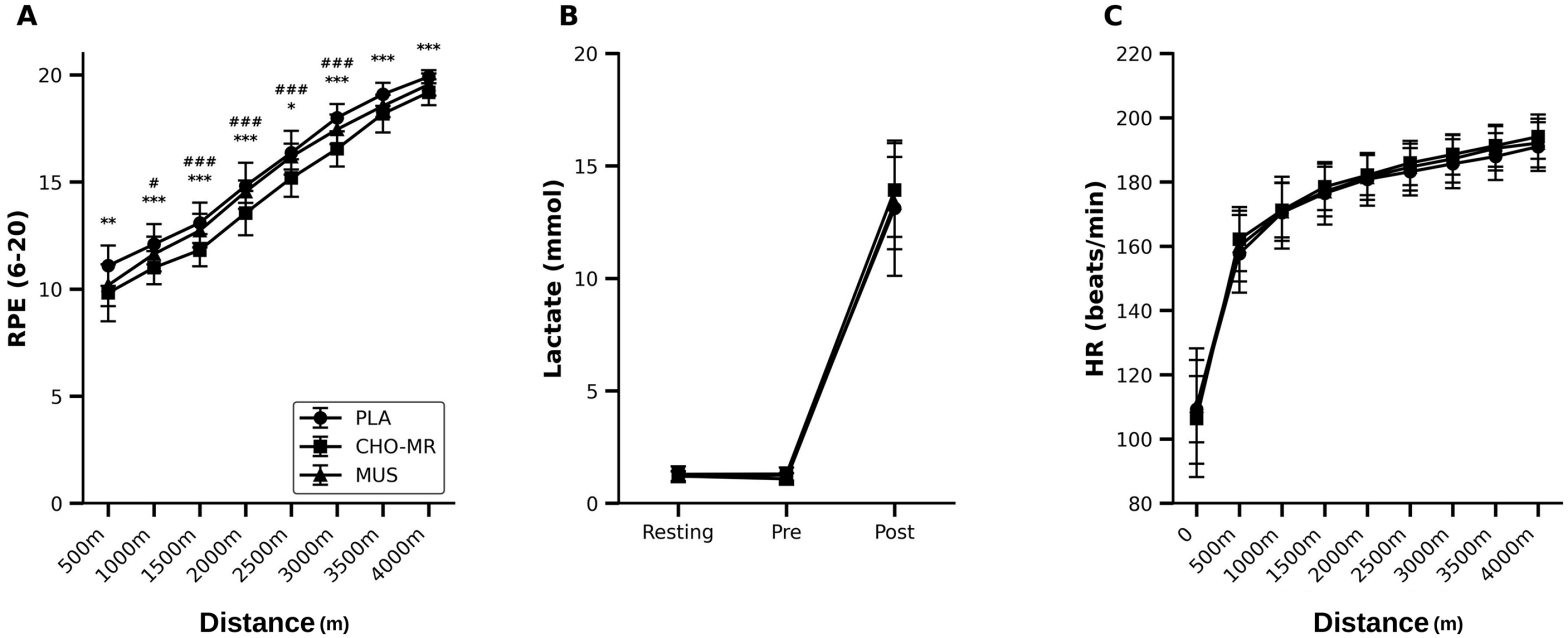
Ratings of perceived exertion, blood lactate concentration, and heart rate during the 4-km cycling time trial under CHO-MR, MUS, and PLA conditions. (A) RPE at 500-m intervals. Significant main effects of condition (*p* < 0.01) and distance (*p* < 0.001) and a condition × distance interaction (*p* < 0.001) were observed. (B) Blood lactate concentration at rest/pre-exercise and post-exercise. A significant main effect of stage was observed (*p* < 0.001) without between-condition differences (*p* ≥ 0.05). (C) HR at 500-m intervals. A significant main effect of distance (*p* < 0.001) and condition × distance interaction (*p* < 0.001) were observed, with no between-condition differences at individual distances (*p* ≥ 0.05). Values are presented as mean ± SD. In panel (A), symbols denote Holm-adjusted pairwise differences between CHO-MR and PLA (* *p* < 0.05, ** *p* < 0.01, *** *p* < 0.001) and between CHO-MR and MUS (# *p* < 0.05, ## *p* < 0.01, ### *p* < 0.001). CHO-MR, carbohydrate mouth rinsing; HR, heart rate; MUS, music listening; PLA, placebo mouth rinse; RPE, rating of perceived exertion.

Blood lactate concentration increased significantly from resting/pre-exercise to post-exercise (main effect of stage, *p* < 0.001), but no main effect of condition or condition × stage interaction was observed (all *p* ≥ 0.05; Fig 8B). Holm-adjusted comparisons confirmed the absence of between-condition differences at any stage (all *p* ≥ 0.05).

### Heart rate

HR increased progressively across the time trial (main effect of main effects of distance, *p* < 0.001) and showed a significant condition × distance interaction (*p* < 0.001), whereas the main effect of condition was not significant (*p* ≥ 0.05; Fig 8C). Despite the significant interaction, Holm-adjusted pairwise comparisons revealed no between-condition differences at any individual distance point (all *p* ≥ 0.05).

## Discussion

In this randomized crossover trial, CHO-MR increased bilateral DLPFC ΔaccHbO₂ both immediately following the intervention and after the 4-km TT, relative to MUS and PLA. CHO-MR was also associated with preserved post-exercise Stroop performance and reduced RPE throughout the time trial. Together, these findings suggest that oral carbohydrate sensing modulates prefrontal cortical activation during high-intensity exercise, with concomitant effects on cognitive performance and perceived effort.

Although direct evidence linking oral carbohydrate exposure to changes in cortical oxygenation remains limited, previous neuroimaging studies demonstrate that carbohydrate-related oral stimuli activate reward- and control-related brain regions, including primary taste cortex, striatum, cingulate cortex, and DLPFC [3,28]. Activation of these networks occurs independently of metabolic substrate delivery and may reflect anticipatory or motivational signaling processes. In this context, CHO-MR may enhance task-related cortical engagement without altering peripheral energy availability, consistent with reports that carbohydrate ingestion influences cerebral metabolism and oxygen utilization [29,30].

Bilateral prefrontal activation exhibited coordinated modulation across conditions, as indicated by the positive association between right and left DLPFC ΔaccHbO₂. Furthermore, increases in DLPFC oxygenation were positively associated with improvements in Stroop performance. These associations are consistent with the established role of the DLPFC in executive control and interference resolution [22,23], suggesting that greater cortical recruitment may support maintenance of cognitive performance under exercise-induced strain. Although the association appeared stronger in the right hemisphere, the present data do not permit definitive conclusions regarding hemispheric specialization.

Within the framework of central fatigue, our findings suggest that sustained bilateral DLPFC activation may represent a compensatory response that helps mitigate fatigue-related cognitive decline. The pattern of results, with somewhat stronger associations in the right hemisphere, is consistent with—but does not establish—potentially greater contribution of the right DLPFC to interference control during the Stroop test. Marcora’s psychobiological model [19] offers a theoretical context for interpreting these findings, proposing that perceived exertion—largely determined by central neural processing rather than peripheral muscle fatigue alone—is primary determinant of endurance performance. In line with this framework, CHO-MR was associated with elevated bilateral DLPFC oxygen saturation, preserved Stroop performance, and reduced perceived exertion, suggesting that modulation of central processes related to effort perception may contribute to performance regulation [31]. Notably, the CHO-MR condition, which exhibited the highest bilateral DLPFC HbO, demonstrated preserved executive performance relative to MUS and PLA, consistent with prior evidence that CHO-MR attenuates exercise-induced executive impairment independent of metabolic substrate availability [5,32]. However, central fatigue was not directly assessed using neurophysiological techniques such as voluntary activation, electromyography-derived motor drive, or superimposed twitch responses. Therefore, the present inferences are limited to cortical oxygenation and perceptual indices (RPE) as indirect correlates of central processes related to fatigue, rather than definitive indices of central fatigue per se.

In addition to the neurophysiological and cognitive benefits, CHO-MR resulted in shorter completion time compared to both PLA and MUS, and greater mean power output relative to MUS. These findings suggest that modulation of prefrontal cortical activity and perceived exertion may translate into measurable performance benefits, even during short-duration, high-intensity exercise. However, the magnitude of improvement was modest (approximately 5–10 s across conditions), and not all performance indices differed consistently between conditions. This variability likely reflects the non-linear relationships among completion time, speed, and power output, as well as inherent variability in time-trial performance. Accordingly, the performance effects should be interpreted as meaningful but moderate. The observed performance improvements align with the psychobiological model of endurance performance [16], which posits that perceived exertion is a primary determinant of exercise tolerance. In the present study, CHO-MR was associated with reduced RPE across the time trial, alongside preserved executive function and elevated DLPFC oxygenation. Together, these findings are consistent with the notion that central modulation of effort perception contributes to pacing and performance regulation. It is plausible that such effects may become more pronounced during longer-duration exercise or repeated efforts, where cumulative central strain plays a greater role in performance limitation [27,28]; however, this remains speculative and warrants direct investigation.

In contrast, music listening did not enhance cycling performance, despite reduced RPE at several distance intervals. This dissociation between perceptual and performance outcomes suggests that reductions in perceived exertion alone may be insufficient to improve high-intensity performance under all conditions. Previous studies report context-dependent ergogenic effects of music, influenced by factors such as prior fatigue, exercise intensity, and individual preference. For example, Lopes-Silva et al. [17] demonstrated altered attentional focus without reversal of fatigue-induced performance decrements during moderate-intensity exercise preceded by cognitive fatigue. The absence of performance improvement with MUS in the present study may therefore reflect the near-maximal intensity of the time trial, limiting the influence of broader psychophysiological modulation. Mechanistically, CHO-MR and MUS likely differ in their neural pathways of action. CHO-MR engages gustatory and reward-related neural circuits via oral carbohydrate receptors, potentially eliciting a more focal activation with the prefrontal and striatal regions. In contrast, MUS operates through distributed networks involving auditory processing, emotional arousal, and attentional regulation. This broader and more variable nature of music-induced modulation may render its ergogenic effects more sensitive to contextual and individual factors.

The absence of between-condition differences in HR and [La⁻] suggests that the performance effects observed with CHO-MR were not accompanied by detectable changes in the measured peripheral physiological variables. Because participants expectorated the carbohydrate solution after each rinse, systemic carbohydrates ingestion was minimized, reducing the likelihood that peripheral metabolic factors accounted for the observed effects. This pattern is consistent with theoretical models proposing that the brain integrates afferent physiological information to regulate effort and performance, including the central governor framework [33,34]. The dissociation between unchanged peripheral markers and improved performance under CHO-MR therefore supports the interpretation that oral carbohydrate sensing may influence exercise regulation through central processes rather than through alterations in systemic metabolism. However, the present data do not exclude subtle peripheral contributions that were not captured by HR or lactate measurements. These findings are broadly consistent with prior studies reporting ergogenic effects of CHO-MR independent of measurable peripheral metabolic changes [1,35].

This study has some limitations. First, the relatively small sample size (n = 11), which is below the a priori target (N = 14), may have reduced statistical power for certain outcomes and limits the generalizability of the findings. Replication in larger cohorts is warranted to confirm these effects and to evaluate CHO-MR efficacy across diverse exercise modalities and populations. Second, the music intervention did not account for individual preferences, which may influence motivational and affective responses to auditory stimuli. Personalized music selection potentially yield different perceptual or performance outcomes [33]. Third, central fatigue was not directly measured using neurophysiological techniques (e.g., voluntary activation or EMG-derived motor drive); thus, inferences regarding central mechanisms are limited to cortical oxygenation and RPE as indirect indices. Fourth, as is inherent to music-exercise research, participant blinding to the MUS condition was not feasible; the CHO-MR and PLA conditions served as no-music comparators within the same crossover design. MUS-related comparisons should therefore be interpreted as exploratory. Additionally, a formal blinding success check for the mouth-rinse conditions was not performed; future studies should include a post-trial guessing questionnaire to verify blinding integrity. Furthermore, participants had access to real-time performance feedback during TT, which may have influenced pacing behaviour; period and carryover effects were statistically adjusted to mitigate this. Fifth, modest inconsistencies observed across performance metrics likely reflect the non-linear relationships between completion time and derived variables (e.g., mean power and speed), as well as device-specific computational algorithms. These findings should therefore be interpreted with appropriate caution. Future research should examine CHO-MR effects across larger and more diverse populations, different exercise modalities and durations, and investigate potential interactions with other ergogenic strategies, including individualized music selection.

## Conclusion

Carbohydrate mouth rinsing was associated with increased bilateral DLPFC oxygenation, preserved executive function, and reduced perceived exertion during high-intensity endurance exercise. These changes were accompanied by modest improvements in time-trial performance without detectable differences in heart rate or blood lactate concentration, consistent with a non-metabolic contribution to performance modulation. In contrast, exploratory comparisons with the music listening condition indicated reduced perceived exertion at selected intervals but no improvement in performance outcomes under the present conditions; these findings should be interpreted with caution given the absence of a matched sham control for the music intervention.

## Supporting information

S1 File Study Protocol. (DOCX)

S2 File CONSORT 2025 Checklist. (DOCX)
