# Supplementary material for: Oral carbohydrate sensing enhances prefrontal cortex oxygenation, reduces perceived exertion, and improves high-intensity cycling performance: A randomized crossover trial
Source: PLoS One. 2026 May 12;21(5):e0349067. doi: 10.1371/journal.pone.0349067 (PMC13166947; doi:10.1371/journal.pone.0349067)
Supplement: S1 File — (DOCX) [file pone.0349067.s001.docx]

**CLINICAL TRIAL PROTOCOL**

ClinicalTrials.gov Identifier: NCT07099807

**Oral carbohydrate sensing enhances prefrontal cortex oxygenation, reduces perceived exertion, and improves high-intensity cycling performance: a randomized crossover trial**

**Study acronym:** CHO-MR-PFC Trial

**Trial registry:** ClinicalTrials.gov

**Registration number:** NCT07099807

**Registration URL:** https://clinicaltrials.gov/study/NCT07099807

**Protocol version:** 1.0

**Protocol date:** April 2025

**IRB approval:** Chung-Ang University Bioethics Committee, approval number 1041078-20250131-BR-024 (8 April 2025)

**Sponsor:** Investigator-initiated; supported by the Ministry of Education of the Republic of Korea and the National Research Foundation of Korea (grant 2025S1A5B5A17013378)

**Principal investigator:** Doug Hyun Han, MD, PhD — Department of Psychiatry, Chung-Ang University Hospital, Seoul 06973, Republic of Korea (hduk70@gmail.com)

**Co-investigators:** Seung-Bo Park; Kyungjin Oh; Geonwoo Yang; Taenam Kim; Jea-Woog Lee; Hyung-Jin Jeon

*Note. This protocol document corresponds to the trial registered as NCT07099807 and reflects the design and procedures actually conducted. Deviations from the originally envisioned study (e.g., omission of the mental fatigue task, exclusion of OFC analyses, removal of the planned functional connectivity analysis, and final completed sample size of n = 11) are described in Section 10.*

# 1. Background and rationale

High-intensity endurance exercise places substantial demands on central neural processes governing effort regulation, motivation, and attentional control. The dorsolateral prefrontal cortex (DLPFC) plays a key integrative role in these processes and exhibits altered hemodynamic responses under fatiguing exercise, which have been linked to elevated perceived exertion and impaired executive performance.

Carbohydrate mouth rinsing (CHO-MR) has been proposed as a non-metabolic ergogenic intervention. Activation of oral carbohydrate receptors stimulates reward- and control-related brain regions, including the DLPFC and striatum, without altering circulating fuel availability. Music listening (MUS) represents a separate non-metabolic intervention thought to modulate central pathways through engagement of prefrontal and mesolimbic networks, with effects on affect, arousal, and ratings of perceived exertion (RPE).

Despite extensive independent investigation of CHO-MR and MUS, direct head-to-head comparisons of their effects on cerebral oxygenation and DLPFC-dependent cognitive performance during high-intensity endurance exercise are scarce. The 4-km cycling time trial (TT) is a near-maximal effort lasting approximately 5–6 minutes; it imposes pronounced cognitive and motivational demands while minimizing exogenous fuel availability, providing a sensitive paradigm for assessing cortical oxygenation and perceptual responses.

# 2. Objectives and hypotheses

## 2.1 Primary objective

To examine the effects of CHO-MR and MUS on bilateral DLPFC oxygenation, executive function, perceived exertion, and 4-km cycling time-trial performance, relative to a placebo mouth rinse (PLA), in well-trained cyclists.

## 2.2 Hypotheses

- H1. Both CHO-MR and MUS will increase DLPFC oxygenation during a cognitive task relative to PLA.
- H2. Both interventions will improve executive performance compared with PLA.
- H3. CHO-MR will sustain greater DLPFC oxygenation during subsequent high-intensity exercise than MUS, indicating differential central modulation independent of peripheral metabolic alterations.

# 3. Trial design

Single-blind, randomized, counterbalanced, three-condition crossover trial (allocation ratio 1:1:1 across the three condition orders within each participant). Framework: superiority. Each participant served as their own control and completed all three conditions in a randomized order, with washout between trials.

# 4. Trial setting

Single-site laboratory study conducted at the Human Performance Laboratory, Graduate School of Sports Medicine, CHA University (Republic of Korea). All sessions were performed in a temperature-controlled laboratory environment.

# 5. Eligibility criteria

## 5.1 Inclusion criteria

- Trained cyclists with ≥2 years of structured endurance cycling training.
- Habitual training volume of ≥5 days/week and approximately 4 h/day.
- Age ≥18 years.
- Provided written informed consent.

## 5.2 Exclusion criteria

- Cardiovascular, neurological, or metabolic disorders.
- Current use of medications known to affect cardiovascular or cognitive function.
- Musculoskeletal injury within the prior 3 months affecting cycling performance.
- Confirmed needle phobia precluding capillary blood sampling.
- Inability to comply with the testing schedule.

# 6. Interventions

## 6.1 Carbohydrate mouth rinse (CHO-MR)

A 6.4% maltodextrin solution (64 g/L; Nutricost LLC, Vineyard, UT, USA) was prepared in distilled water. During each session, participants performed five standardized rinses of 25 mL each. For each rinse, the solution was swilled throughout the oral cavity for 10 s and then expectorated; rinses were separated by 30-s intervals. Participants were instructed not to swallow any solution.

## 6.2 Music listening (MUS)

High-tempo music at 120 beats per minute was delivered for 15 min via standardized in-ear earbuds connected to a smartphone. The playlist ("120 BPM Best Dance Music for Running and Working Out", publicly available on YouTube) was identical for all participants. Sound intensity was calibrated to approximately 65 dB (≈50% of maximum device output) according to manufacturer specifications.

## 6.3 Placebo mouth rinse (PLA)

Distilled water containing approximately 0.05 g/L of non-caloric sucralose (Shaanxi Hongda Phytochemistry Co., Ltd., Xi'an, China) to mimic the subtle sweetness of the maltodextrin solution. Volume, duration, frequency, and rinse procedure were identical to CHO-MR. Both solutions were colorless and presented in opaque containers to support blinding.

## 6.4 Adherence and concomitant care

Interventions were delivered by trained study staff under direct observation; full adherence was achieved for all 33 sessions (11 participants × 3 conditions). Participants were instructed to maintain habitual training and to refrain from new dietary supplements during the trial period. No concomitant care was provided as part of the trial.

# 7. Outcomes

## 7.1 Primary outcome

Bilateral DLPFC accumulated oxygenated hemoglobin concentration change (ΔaccHbO₂), measured by functional near-infrared spectroscopy (fNIRS) at predefined time points: rest, baseline (Stroop), post-intervention, and post-4-km TT. Measurement variable: ΔHbO₂ relative to resting baseline. Method of aggregation: mean across channels within each region of interest, then mean across participants per condition × stage.

## 7.2 Secondary outcomes

- Stroop test performance (executive function): score recorded at baseline, post-intervention, and post-TT.
- Rating of perceived exertion (RPE): Borg 6–20 scale at 500-m intervals during the 4-km TT.
- Cycling performance: completion time (s, primary performance variable), mean power output (W), peak power output (W), and mean speed (km/h).
- Heart rate (HR): continuous monitoring; values recorded at 500-m intervals.
- Capillary blood lactate concentration ([La⁻]): pre-exercise (post-warm-up) and immediately post-TT.

## 7.3 Harms

Adverse events were monitored throughout each session by study staff. The protocol involved minimal-risk physiological monitoring; the only anticipated minor risk was localized bruising from earlobe capillary sampling. No adverse events were observed across the trial.

# 8. Sample size

An a priori sample-size estimation was performed using G*Power 3.1 (Heinrich Heine University Düsseldorf, Düsseldorf, Germany) based on a repeated-measures ANOVA framework (three within-subject conditions). Assumptions: effect size f = 0.36, α = 0.05, power (1−β) = 0.80, correlation among repeated measures r = 0.50, and ε = 1.0. The required sample size was N = 14 completers; therefore, N = 15 was targeted to allow for potential attrition. No interim analyses or stopping rules were planned.

Following adoption of generalized estimating equations (GEE) as the analytic model with adjustment for crossover-related effects, statistical power was additionally evaluated via Monte Carlo simulation (3,000 replicates) matched to the observed data structure (11 participants, three conditions, four stages). Estimated power for the global condition × stage interaction and for the Holm-adjusted CHO-MR versus PLA contrast at post-TT ranged from 0.67 to 0.92 across hemispheres.

# 9. Randomization, allocation concealment, and blinding

## 9.1 Sequence generation

The order of the three experimental conditions (CHO-MR, MUS, PLA) was randomized and counterbalanced using a computer-generated allocation sequence to minimize sequence and order effects.

## 9.2 Allocation concealment

Mouth-rinse solutions were prepared in advance by personnel not involved in outcome assessment and presented in opaque, identical containers labelled by trial code. Participants did not have visual or olfactory cues to distinguish CHO-MR from PLA.

## 9.3 Blinding

Single-blind: participants were blinded to the identity of the mouth-rinse solution (CHO-MR vs PLA). Owing to the perceptible nature of the music intervention, full blinding to MUS was not feasible. Outcome assessors involved in fNIRS preprocessing were blinded to condition labels.

# 10. Trial schedule and changes from the originally envisioned design

The trial comprised five laboratory visits per participant:

- Visit 1: medical screening, anthropometry, and incremental cycling test to determine functional threshold power (FTP).
- Visit 2: full familiarization, including a 4-km TT, mouth-rinsing procedures, cognitive testing, and physiological monitoring.
- Visits 3–5: experimental trials (CHO-MR, MUS, PLA), with order randomized and counterbalanced. A washout period of 3–7 days separated experimental trials. All sessions for a given participant were scheduled at the same time of day.

## 10.1 Pre-specified deviations

The following modifications, finalized prior to statistical analysis, are noted for transparency:

- Final sample size: 11 completers (vs target N = 15) due to participant withdrawal and scheduling constraints.
- A mental fatigue induction task originally considered prior to intervention was omitted to reduce participant burden; the Stroop test was retained as a brief probe of executive function with concurrent fNIRS measurement, not as a fatigue-induction protocol.
- Planned analyses of functional connectivity were not performed due to technical limitations during fNIRS data acquisition; analyses focused on regional ΔaccHbO₂ as pre-specified.
- Outcome focus: bilateral DLPFC ΔaccHbO₂ was retained as the primary outcome; orbitofrontal, frontopolar, and ventrolateral prefrontal regions were included in the channel layout but not the primary analysis.
- Trial registration was performed retrospectively on ClinicalTrials.gov (NCT07099807).

# 11. Procedures and measurements

## 11.1 Anthropometry and FTP

Body mass and stature were obtained using standardized procedures. An incremental cycling test was performed on a Wattbike Pro ergometer (Wattbike Ltd., Nottingham, UK) following a standardized warm-up (5 min self-selected moderate intensity, then 3 min at ≈50% of anticipated maximal workload). The incremental protocol began at 100 W, with workload increased by 15 W/min for female cyclists and 20 W/min for male cyclists. Cadence was maintained ≥60 rpm. The test ended when cadence fell below 60 rpm despite verbal encouragement. FTP was estimated as 75% of the peak power output achieved during the final completed stage.

## 11.2 Experimental session sequence

Each experimental session followed an identical sequence: (i) 3-min seated rest with concurrent fNIRS recording; (ii) 3-min Stroop task with fNIRS; (iii) 15-min intervention (CHO-MR, MUS, or PLA); (iv) 3-min Stroop task with fNIRS; (v) capillary [La⁻] sampling; (vi) standardized warm-up (10 min cycling at 60% VO₂max followed by 5 min passive recovery); (vii) pre-exercise [La⁻]; (viii) 4-km cycling TT with continuous HR and 500-m RPE; (ix) immediate post-TT [La⁻]; (x) 3-min Stroop with concurrent fNIRS.

## 11.3 fNIRS acquisition and preprocessing

Cortical hemodynamic response was assessed using a high-density continuous-wave fNIRS system (NIRSIT; OBELAB Inc., Seoul, Korea). The system has 24 dual-wavelength sources (780 and 850 nm) and 32 photodetectors, sampled at 8.138 Hz, with source–detector separations of 15–30 mm. Only 30-mm channels were retained for primary analysis. Raw optical density signals were converted to ΔHbO and ΔHbR using the modified Beer–Lambert law and band-pass filtered (0.01–0.10 Hz). Channels with signal-to-noise ratio < 30 dB were excluded. Primary analyses focused on bilateral DLPFC channels (right: 1, 2, 3, 5, 6, 11, 17, 18; left: 19, 20, 33, 34, 35, 38, 39, 43).

## 11.4 Stroop task

A 3-min computerized Stroop color–word task was administered on a Windows-based tablet. Each task comprised congruent and incongruent stimuli; participants identified the ink color while inhibiting the automatic tendency to read the word. A familiarization session preceded experimental trials.

## 11.5 4-km cycling time trial

Performed on a calibrated Wattbike Pro ergometer. Initial pacing guidance suggested ≈105–110% of FTP; pacing was self-selected. Real-time feedback (distance, instantaneous power, elapsed time) was displayed; standardized verbal encouragement was provided. RPE was recorded at 500-m intervals using the Borg 6–20 scale. HR was monitored continuously using a Polar H10 sensor (Polar Electro, Kempele, Finland).

## 11.6 Capillary blood lactate

20 µL of capillary blood was obtained from the earlobe using a single-use lancet, mixed in hemolysis solution, and analyzed using an amperometry–enzymatic method (Biosen C-line, EKF Diagnostics GmbH, Barleben, Germany).

# 12. Statistical analysis plan

All analyses were performed using Python (v3.13.3) with the statsmodels (v0.14.5) and SciPy (v1.15.3) packages. Significance was set at α = 0.05 (two-tailed); 95% confidence intervals were reported where appropriate.

## 12.1 Primary analysis

Bilateral DLPFC ΔaccHbO₂ was modelled using generalized estimating equations (GEE) with a Gaussian family, exchangeable working correlation structure, and robust (sandwich) standard errors. Fixed effects: condition (CHO-MR, MUS, PLA), stage (resting, baseline, post-intervention, post-TT), and condition × stage interaction. Models were adjusted for period (numeric), sequence, and first-order carryover (previous condition). Global robust Wald χ² tests evaluated main effects and interactions. Where significant, estimated marginal means and Holm-corrected pairwise contrasts were derived.

## 12.2 Secondary analyses

- Cycling performance outcomes (completion time, mean power, peak power, mean speed): GEE with condition as the predictor and adjustment for period, sequence, and first-order carryover.
- RPE: GEE with distance treated as the repeated stage factor; Holm-adjusted pairwise contrasts at each distance.
- Stroop performance: GEE analogous to the primary fNIRS model.
- Associations between post-exercise change scores in DLPFC ΔaccHbO₂ and ΔStroop performance: GEE regression adjusted for period, sequence, and first-order carryover.

## 12.3 Handling of missing data

All 11 participants completed all three conditions. GEE accommodates within-subject correlation; complete-case analysis was used because no participants had missing within-condition outcome data.

# 13. Ethics, consent, and dissemination

The protocol was approved by the Chung-Ang University Bioethics Committee (approval number 1041078-20250131-BR-024) and was conducted in accordance with the Declaration of Helsinki. Written informed consent was obtained from all participants prior to enrolment. Personal data were stored securely in offline or encrypted form; physical records were sealed and retained for 3 years before secure destruction. Withdrawal of consent at any point would result in immediate destruction of the participant's data.

# 14. Funding and competing interests

This work was supported by the Ministry of Education of the Republic of Korea and the National Research Foundation of Korea (grant 2025S1A5B5A17013378). The funder had no role in study design, data collection and analysis, decision to publish, or preparation of the manuscript. The authors declare that no competing interests exist.

# 15. Data availability

De-identified individual participant data and analysis code supporting the findings of this trial are available from the corresponding author (Doug Hyun Han, hduk70@gmail.com) upon reasonable request, subject to applicable institutional and ethical approvals.

# 16. Trial status

Recruitment period: 17–27 April 2025. Data collection has been completed; the trial is closed to recruitment. Trial registration on ClinicalTrials.gov (NCT07099807) was performed retrospectively.
