# Supplementary material for: Oral carbohydrate sensing enhances prefrontal cortex oxygenation, reduces perceived exertion, and improves high-intensity cycling performance: A randomized crossover trial
Source: PLoS One. 2026 May 12;21(5):e0349067. doi: 10.1371/journal.pone.0349067 (PMC13166947; doi:10.1371/journal.pone.0349067)
Supplement: S2 File — (DOCX) [file pone.0349067.s002.docx]

**CONSORT 2025 Checklist**

*Information to include when reporting a randomized trial*

**Manuscript title:** Oral carbohydrate sensing enhances prefrontal cortex oxygenation, reduces perceived exertion, and improves high-intensity cycling performance: a randomized crossover trial

**Manuscript ID:** PONE-D-25-36197R2

**Clinical trial registration:** ClinicalTrials.gov Identifier NCT07099807

**Corresponding author:** Doug Hyun Han (hduk70@gmail.com)

| **Section/Topic** | **Item No** | **CONSORT 2025 Checklist item** | **Reported on page No / Section** |
| --- | --- | --- | --- |
| **Title and abstract** | | | |
| Title and structured abstract | **1a** | Identification as a randomized trial | Title page ("randomized crossover trial" in title) |
|  | **1b** | Structured summary of the trial design, methods, results, and conclusions | Abstract (Purpose, Methods, Results, Conclusion) |
| **Open science** | | | |
| Trial registration | **2** | Name of trial registry, identifying number (with URL) and date of registration | Title page; Methods – Participants (ClinicalTrials.gov NCT07099807; https://clinicaltrials.gov/study/NCT07099807) |
| Protocol and statistical analysis plan | **3** | Where the trial protocol and statistical analysis plan can be accessed | Supporting Information (S1 File – Study Protocol) |
| Data sharing | **4** | Where and how the individual de-identified participant data, statistical code and any other materials can be accessed | Data Availability Statement (available from corresponding author upon reasonable request) |
| Funding and conflicts of interest | **5a** | Sources of funding and other support, and role of funders in the design, conduct, analysis and reporting of the trial | Financial Disclosure (Ministry of Education / NRF Korea, 2025S1A5B5A17013378; funder had no role) |
|  | **5b** | Financial and other conflicts of interest of the manuscript authors | Competing Interests statement (none declared) |
| **Introduction** | | | |
| Background and rationale | **6** | Scientific background and rationale | Introduction (paragraphs 1–3) |
| Objectives | **7** | Specific objectives related to benefits and harms | Introduction (final paragraph – three a priori hypotheses) |
| **Methods** | | | |
| Patient and public involvement | **8** | Details of patient or public involvement in the design, conduct and reporting of the trial | Not applicable – participants were not involved in the design or analysis |
| Trial design | **9** | Description of trial design including type of trial, allocation ratio, and framework | Methods – Study design (single-blind, randomized, counterbalanced 1:1:1 crossover; superiority framework) |
| Changes to trial protocol | **10** | Important changes to the trial after it commenced including any outcomes or analyses that were not prespecified, with reason | Methods – Participants (target N=15→11 completers; mental fatigue task omitted; planned functional connectivity analysis not performed due to technical limitations) |
| Trial setting | **11** | Settings and locations where the trial was conducted | Methods – Participants (single-site laboratory study; Republic of Korea) |
| Eligibility criteria | **12a** | Eligibility criteria for participants | Methods – Participants (≥2 years endurance cycling; no cardiovascular/neurological/metabolic disorders; no relevant medications) |
|  | **12b** | If applicable, eligibility criteria for sites and for individuals delivering the interventions | Not applicable – single-site trial; standardized interventions delivered by study staff |
| Intervention and comparator | **13** | Intervention and comparator with sufficient details to allow replication | Methods – Carbohydrate mouth rinse; Music intervention (CHO-MR 6.4% maltodextrin; MUS 120 bpm; PLA sucralose ≈0.05 g/L) |
| Outcomes | **14** | Prespecified primary and secondary outcomes, including measurement variable, analysis metric, method of aggregation, and time point | Methods – Study design; Assessment of hemodynamic changes (primary: bilateral DLPFC ΔaccHbO₂; secondary: Stroop, RPE, completion time, power, HR, [La⁻]) |
| Harms | **15** | How harms were defined and assessed (e.g., systematically, non-systematically) | Methods – 4-km cycling time trial; Discussion (no adverse events were observed; minimal-risk physiological monitoring) |
| Sample size | **16a** | How sample size was determined, including all assumptions supporting the sample size calculation | Methods – Sample Size and Power Analysis (G*Power 3.1; f=0.36, α=0.05, 1-β=0.80, r=0.50; N=14 required, target 15; supplemented by Monte Carlo power simulation) |
|  | **16b** | Explanation of any interim analyses and stopping guidelines | Not applicable – no interim analyses planned or performed |
| Randomization: Sequence generation | **17a** | Who generated the random allocation sequence and the method used | Methods – Study design (computer-generated allocation sequence) |
|  | **17b** | Type of randomization and details of any restriction | Methods – Study design (counterbalanced crossover; condition order randomized) |
| Allocation concealment mechanism | **18** | Mechanism used to implement the random allocation sequence | Methods – Study design and Carbohydrate mouth rinse (opaque, identical containers prepared in advance) |
| Implementation | **19** | Whether the personnel who enrolled and those who assigned participants to the interventions had access to the random allocation sequence | Methods – Study design (allocation sequence administered independently of enrolment) |
| Blinding | **20a** | Who was blinded after assignment to interventions | Methods – Study design and Carbohydrate mouth rinse (single-blind: participants blinded to CHO-MR vs PLA; MUS not blindable) |
|  | **20b** | If blinded, how blinding was achieved and description of the similarity of interventions | Methods – Carbohydrate mouth rinse (colorless solutions in opaque containers; matched volume, duration, frequency); Discussion – Limitations (no formal blinding-success check) |
| Statistical methods | **21a** | Statistical methods used to compare groups for primary and secondary outcomes, including harms | Methods – Statistical analyses (GEE with Gaussian family, exchangeable correlation, robust SE; condition × stage models) |
|  | **21b** | Definition of who is included in each analysis, and in which group | Methods – Statistical analyses; Results (all 11 completers analyzed under each crossover condition) |
|  | **21c** | How missing data were handled in the analysis | Methods – Statistical analyses (GEE accommodates within-subject correlation; complete-case analysis as no participants had missing within-condition data) |
|  | **21d** | Methods for any additional analyses, distinguishing prespecified from post hoc | Methods – Statistical analyses (Holm-adjusted pairwise EMM contrasts; pooled change-score GEE associations) |
| **Results** | | | |
| Participant flow, including flow diagram | **22a** | For each group, the numbers of participants who were randomly assigned, received intended intervention, and were analyzed for the primary outcome | Methods – Participants; Fig 1 – Flowchart (n=11 enrolled, randomized, completed all three conditions, included in analysis) |
|  | **22b** | For each group, losses and exclusions after randomization, together with reasons | Methods – Participants (4 of original target N=15 not completed: withdrawal and scheduling constraints) |
| Recruitment | **23a** | Dates defining the periods of recruitment and follow-up for outcomes of benefits and harms | Methods – Participants (recruited 17–27 April 2025) |
|  | **23b** | If relevant, why the trial ended or was stopped | Not applicable – trial ended as planned upon completion of recruitment target |
| Intervention and comparator delivery | **24a** | Intervention and comparator as they were actually administered | Methods – Carbohydrate mouth rinse; Music intervention; Experimental protocol (delivered by trained staff per protocol; full adherence) |
|  | **24b** | Concomitant care received during the trial for each group | Not applicable – no concomitant care; participants instructed to maintain habitual training and refrain from supplements during trial period |
| Baseline data | **25** | A table showing baseline demographic and clinical characteristics for each group | Table 1 – Participant main characteristics and performance details |
| Numbers analyzed, outcomes and estimation | **26** | For each primary and secondary outcome, by group: number analyzed, number with available data, result for each group, estimated effect size and its precision | Results – Changes in DLPFC across conditions; Stroop performance; Performance outcomes; Ratings of perceived exertion; Physiological parameters (Figs 4–8; n=11 per condition; effect sizes reported with robust Wald χ², z statistics and 95% CIs as appropriate) |
| Harms | **27** | All harms or unintended events in each group | Methods – 4-km cycling time trial; no adverse events were reported under any condition |
| Ancillary analyses | **28** | Any other analyses performed, including subgroup and sensitivity analyses, distinguishing pre-specified from post hoc | Methods – Statistical analyses; Results – Associations between ΔaccHbO₂ and Stroop (pooled change-score GEE; pre-specified exploratory) |
| **Discussion** | | | |
| Interpretation | **29** | Interpretation consistent with results, balancing benefits and harms, and considering other relevant evidence | Discussion (paragraphs 1–4) |
| Limitations | **30** | Trial limitations, addressing sources of potential bias, imprecision, generalizability, and, if relevant, multiplicity of analyses | Discussion – Limitations paragraph (sample size, MUS blinding, no direct central fatigue measure, single population) |

*Reference: Hopewell S, Chan A-W, Collins GS, Hróbjartsson A, Moher D, Schulz KF, et al. CONSORT 2025 statement: updated guideline for reporting randomized trials. PLoS Med. 2025;22(4):e1004587. https://doi.org/10.1371/journal.pmed.1004587*

*Page numbers refer to the manuscript file PONE-D-25-36197R2_FTC.docx. Section names refer to headings within the same manuscript.*
